# A New Water-Soluble Bactericidal Agent for the Treatment of Infections Caused by Gram-Positive and Gram-Negative Bacterial Strains

**DOI:** 10.3390/antibiotics9090586

**Published:** 2020-09-08

**Authors:** Alessandro Presentato, Elena Piacenza, Antonino Scurria, Lorenzo Albanese, Federica Zabini, Francesco Meneguzzo, Domenico Nuzzo, Mario Pagliaro, Delia Chillura Martino, Rosa Alduina, Rosaria Ciriminna

**Affiliations:** 1 Department of Biological, Chemical, and Pharmaceutical Science and Technology (STEBICEF), University of Palermo, Viale delle Scienze, 90128 Palermo, Italy; alessandro.presentato@unipa.it (A.P.); elena.piacenza91@gmail.com (E.P.); delia.chilluramartino@unipa.it (D.C.M.); 2 National Interuniversity Consortium of Materials Science and Technology (INSTM), Via G. Giusti 9, 50121 Firenze, Italy; 3 Istituto per lo Studio dei Materiali Nanostrutturati, CNR, via U. La Malfa 153, 90146 Palermo, Italy; antonino.scurria@ismn.cnr.it (A.S.); rosaria.ciriminna@cnr.it (R.C.); 4 Istituto per la Bioeconomia, CNR, via Madonna del Piano 10, 50019 Sesto Fiorentino, FI, Italy; lorenzo.albanese@cnr.it (L.A.); federica.zabini@cnr.it (F.Z.); francesco.meneguzzo@cnr.it (F.M.); 5 Istituto per la Ricerca e l’Innovazione Biomedica, CNR, via U. La Malfa 153, 90146 Palermo, Italy; domenico.nuzzo@cnr.it

**Keywords:** citrus pectin, lemon, grapefruit, IntegroPectin, flavonoids, citrus terpenes, polyphenols, antimicrobial resistance, *Staphylococcus aureus*, *Pseudomonas aeruginosa*

## Abstract

Grapefruit and lemon pectin obtained from the respective waste citrus peels via hydrodynamic cavitation in water only are powerful, broad-scope antimicrobials against Gram-negative and -positive bacteria. Dubbed IntegroPectin, these pectic polymers functionalized with citrus flavonoids and terpenes show superior antimicrobial activity when compared to commercial citrus pectin. Similar to commercial pectin, lemon IntegroPectin determined ca. 3-log reduction in *Staphylococcus aureus* cells, while an enhanced activity of commercial citrus pectin was detected in the case of *Pseudomonas aeruginosa* cells with a minimal bactericidal concentration (MBC) of 15 mg mL^−1^. Although grapefruit and lemon IntegroPectin share equal MBC in the case of *P. aeruginosa* cells, grapefruit IntegroPectin shows boosted activity upon exposure of *S. aureus* cells with a 40 mg mL^−1^ biopolymer concentration affording complete killing of the bacterial cells. Insights into the mechanism of action of these biocompatible antimicrobials and their effect on bacterial cells, at the morphological level, were obtained indirectly through Fourier Transform Infrared spectroscopy and directly through scanning electron microscopy. In the era of antimicrobial resistance, these results are of great societal and sanitary relevance since citrus IntegroPectin biomaterials are also devoid of cytotoxic activity, as already shown for lemon IntegroPectin, opening the route to the development of new medical treatments of polymicrobial infections unlikely to develop drug resistance.

## 1. Introduction

Causing annually 700,000 deaths worldwide [1], antimicrobial resistance (AMR) represents one of today’s main societal burdens in both developed and developing countries. A recent estimate of the implications and costs of AMR assessed more than EUR 9 billion and USD 20 billion to be the economic costs due to clinical interventions and productivity losses [2]. Current projections indicate that by 2050 the annual death toll could reach 10 million people, with an economic impact comparable to the 2008 financial crisis [3]. Intense research activities aimed to find or synthesize new efficacious antimicrobials to counteract AMR infections are carried out worldwide in public research centers and pharmaceutical and biotechnology companies [4].

Among natural antimicrobials, pectic polysaccharides have been known since the 1930s to exert antibacterial activity [5]. However, the almost concomitant introduction and subsequent widespread use of antibiotics led to ignoring this remarkable property of pectins until the late 1990s [6], leaving unveiled, for the most part, their mechanism(s) of action and their potential for different applications in medicine and pharmacology [7,8,9].

Widely employed in the food, medical, and pharmaceutical industries due to its unique structural and biological properties [10,11], pectin is the collective name given to the branched polysaccharides industrially extracted from biological resources such as waste citrus peel, apple pomace, and (to a minor extent) sugar beet. Key structural parameters of pectins such as the degree of esterification, the molecular weight, and the relative proportion of homogalacutonan (HG) “smooth” regions (6-methylated and 2- and/or 3-acetylated poly-α(1–4)-D-galacturonic acid residues), alternating with branched α(1–2)-l-rhamnosyl-α(1–4)-D-galacturonosyl chains substituted with side chains of mainly α-L-arabinofuranose and α-D-galactopyranose (known as rhamnogalacturonan I, RG-I, “hairy” regions), affect both the rheological properties in water and the biological properties including antimicrobial, antioxidant, anticancer, anti-inflammatory, and immunoregulatory action [8].

We have recently reported that lemon pectin derived from waste lemon peels via hydrodynamic cavitation (henceforth referred to as lemon IntegroPectin) exhibits strong in vitro activity against the indicator bacterial *Staphylococcus aureus* strain [12]. *S. aureus* is an opportunistic bacterial pathogen and is the primary cause of infective endocarditis and prosthetic implant-related infections [13]. This bacterial strain is also responsible for bacteremia, skin, and soft tissue clinical infections, with a high mortality rate globally [13]. Beyond clinical settings, Gram-positive *S. aureus* causes infections in ruminants such as cattle, goats, and sheep, leading to clinical and subclinical mastitis, representing a major economic problem for farmers and the dairy industry [14,15]. Similarly, *Pseudomonas aeruginosa* is a bacterial strain virtually found in almost every environmental niche due to its metabolic versatility and ability to degrade complex xenobiotics of anthropogenic origin [16]. This Gram-negative strain is also an opportunistic bacterial pathogen able to induce serious infections threatening human health [17]. For example, *P. aeruginosa* is one of the four frequently encountered bacterial strains responsible for causing hospital-acquired pneumonia, due to its ability to grow as a biofilm on endotracheal tubes in intubated patients, causing an accelerated decline of pulmonary function in cystic fibrosis patients [18]. Furthermore, this strain is also responsible for infections in surgical sites, chronic decubitus ulcers, and in the urinary tract [19]. Unfortunately, *P. aeruginosa* exhibits significant resistance to both innate immune antimicrobial peptides and several antibiotics [18].

To manage *P. aeruginosa* infections, causing high mortality in critically ill and immunocompromised patients driven by the appearance of drug-resistant strains, today’s therapeutic options include antibiotic combinations based on pharmacokinetic and pharmacodynamic analyses [20]. Similarly, several new agents for the treatment of methicillin-resistant *S. aureus* (MRSA) are currently undergoing clinical trials, although these data in real-life terms are limited and require further investigations [21]. Moreover, the increase in antibiotic-resistant phenotypes and antibiotic resistance genes in different environments [22] and widely different organisms [23] requires urgent identification and development, at an industrial level, of new antimicrobial solutions. In this context, highly desirable new anti-pseudomonal and -staphylococcal antimicrobials should not drive superinfection [20] and be preferably available as oral formulations to allow step-down therapy in the treatment of both Gram-negative and -positive infections [24].

Beyond describing and quantifying the high in vitro antibacterial activity of both lemon and grapefruit IntegroPectin against *S. aureus* and *P. aeruginosa* bacterial strains in comparison to that shown by commercial citrus pectin, this study offers the first insight into the mechanism(s) of action of these new natural antimicrobials derived from agri-food industry by-products directly on semi-industrial scale [11], based on structural investigation via Fourier transform infrared (FTIR) spectroscopy and bacterial morphological changes detected by scanning electron microscopy (SEM).

## 2. Results and Discussion

**Figure 1:**
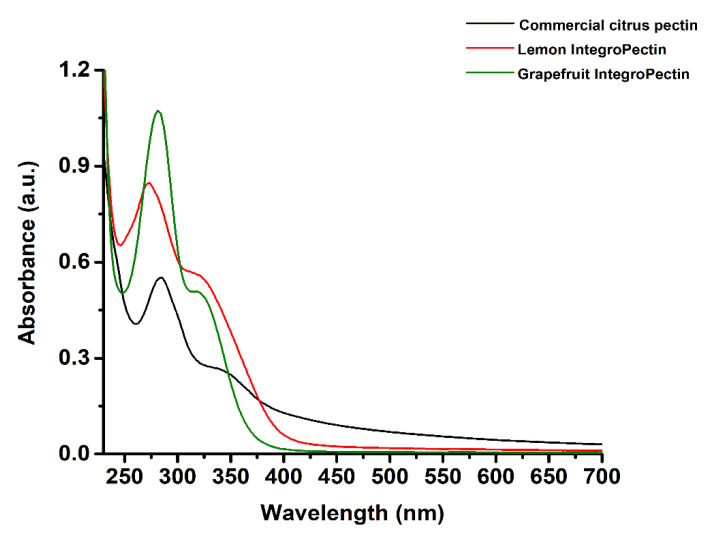
Absorbance spectra of commercial citrus pectin, lemon, and grapefruit IntegroPectins.

**Figure 2:**
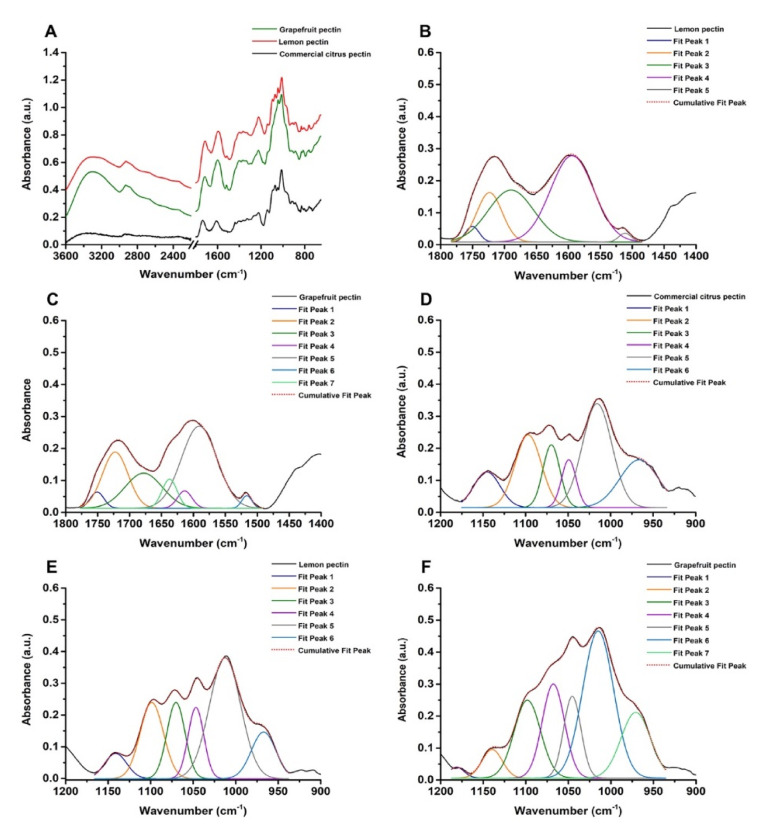
Attenuated total reflectance (ATR)-FTIR spectra collected for lemon and grapefruit IntegroPectin, as well as commercial citrus pectin (**A**) off-set by 0.2 a.u.; spectral deconvolution by non-linear least-squares fitting of lemon (**B**) and grapefruit (**C**) IntegroPectin in the 1800–1470 cm^−1^ region, alongside commercial citrus pectin (**D**), lemon (**E**) and grapefruit (**F**) IntegroPectin in the 1200–950 cm^−1^ region.

The IntegroPectins used throughout this study were extracted via hydrodynamic cavitation (HC) of citrus wastes consisting of either waste lemon or grapefruit peels in water only followed by lyophilization. The process used for lemon IntegroPectin [10] was extended to grapefruit peels. The waste citrus peels, which include in each case residues of the seeds, were kindly donated by a citrus juice factory located in Sicily (Campisi Italia Agricoltura Biologica). In each case, all lemons and grapefruits originating the peels were organically grown (i.e., no agrochemical residues were present in the pectin raw materials). Since the structure and composition of pectins strongly influence their physical–chemical features and applicative properties [25], UV-visible (Figure 1) and FTIR spectroscopies in attenuated total reflectance (ATR) mode (Figure 2) were performed on lemon and grapefruit IntegroPectin, using commercial citrus pectin as a comparison.

The three pectic samples featured the main absorbance peak centered between 273 (Lemon IntegroPectin) and 282 nm (commercial citrus pectin and grapefruit IntegroPectin), as well as a shoulder at ca. 320 (the two IntegroPectins) or 350 nm (commercial citrus pectin) (Figure 1). These absorbance signals are attributable to organic substances, such as polyphenols (ca. 280 nm), flavonoids (ca. 325 nm), and flavonols (ca. 350 nm) [26,27], which, depending on the extraction procedure used, can be found as “impurities” in a small amount within the final pectic extract [28]. However, the higher absorbance detected in the case of lemon and grapefruit IntegroPectins as compared to the commercial citrus pectin suggests the presence of a high amount of polyphenols and flavonoids in the extracts, likely preserved by the HC-based process used for IntegroPectin recovery. Indeed, this process prevents the loss of volatile and less stable compounds, which are lost via the conventional acidic hydrolysis of citrus peel in hot water followed by precipitation of pectin with isopropyl alcohol used to manufacture the commercial pectin. Moreover, absorbance peaks at 280 and 320 nm are typically detected for phenolic acids abundantly present in citrus fruits (e.g., hydroxycinnamic acids) [29]. 

ATR-FTIR spectroscopy allowed to finely unveil the nature of the pectic material utilized in this study. Full band assignments (observed maxima) are reported in Table S1. 

Both lemon and grapefruit IntegroPectin show a broad and intense IR absorption band centered at ca. 3300 cm^−1^ corresponding to -OH stretching partially deriving from hydroxyl groups of polysaccharides (e.g., pyranose) and adsorbed water. For comparison, this contribution was less pronounced in the case of commercial citrus pectin (Figure 2A; Table S1), suggesting the co-presence in lemon and grapefruit IntegroPectin polymers of molecules containing a large number of -OH groups, such as polyphenols and flavonoids, abundant in the citrus peel [30]. A partial overlap was observed between the contributions of -OH and -CH_x_ stretching (Figure 2A), since only the IR absorbance (at ca. 2930 cm^−1^) deriving from the asymmetric -CH stretching of CH and CH_3_ groups within the pectin backbone [10,25], as well as CH_2_ moieties of arabinose and galactose of pectins’ “hairy regions” (RG-I) [10], was identified. Pectin backbone vibrational modes were also detected by IR contributions observed at ca. 2865 cm^−1^ related to the symmetric -CH stretching of CH_3_ groups, partly overlapping with the signal due to -CH stretching of pyranose rings [10]. Less intense IR contributions were identified in the 2675–2500 cm^−1^ region (Figure 2A), which were assigned to -OH stretching vibration typical of free carboxylic acids forming dimers [10,25].

The 1800–1400 cm^−1^ region of ATR-FTIR spectra featured two main absorbance peaks between 1750 and 1600 cm^−1^ where IR vibrational modes of C = O mostly contribute [10], as well as a broader signal deriving from pectin rings’ vibration and deformation modes (1400–1300 cm^−1^; Figure 2A). The maximum characteristic of esterified carboxylic groups (i.e., 1760–1730 cm^−1^) [10,25,31,32,33,34] was detected (1736 cm^−1^) only for the commercial citrus pectin (Table S1), due to the high presence (≥74.0%) of galacturonic acid residues. This contribution was partially overlapped in lemon and grapefruit IntegroPectin samples by the large C = O stretching centered at ca. 1713 cm^−1^ (Table S1) of carboxylate and nonconjugated keto groups generally found in aromatic molecules (e.g., carotenoids, phenols, flavonoids, and terpenoids) [30,35,36]. Similarly, the commercial citrus pectin revealed a strong signal for the asymmetric -COO stretching (ca. 1610 cm^−1^; Table S1) of carboxylate groups within polygalacturonic acid [10,34,37,38], which was hidden, for the two IntegroPectins, by the large IR absorbance at 1594–1599 cm^−1^ (Table S1) attributable to aromatic skeleton vibrations [30,36,39,40]. Additional IR absorbance bands related to aromatic compounds, such as carotenoids and phenols [35,36,39,40], were observed in the 1520–1510 cm^−1^ region of lemon and grapefruit IntegroPectins. These bands were not observed in the commercial citrus pectin spectrum (Table S1) reinforcing the hypothesis that the aforementioned molecules occur only in the citrus IntegroPectin samples.

To gain more insights on differences and similarities in terms of structure and composition between the two IntegroPectins, a deconvolution of the IR absorbance registered in the 1800–1470 cm^−1^ region of the IR spectrum was performed (Figure 2B,C; Table S2), highlighting a higher variability of the grapefruit IntegroPectin as compared to the lemon one. The spectral deconvolution of both IntegroPectins showed IR bands comparable in width and integrated area centered at ca. 1750 cm^−1^ deriving from esterified carboxylic groups of galacturonic acid (GalA) of the pectic polymers [10,25,31,32,33,34,38]. This outcome suggests the presence of GalA within the two pectic polymers in a similar percentage. Furthermore, the IR absorption maximum at 1713–1715 cm^−1^ (Table S1) slightly shifted towards longer wavenumbers (i.e., 1722–1724 cm^−1^) in the deconvoluted spectra (Figure 2B,C; Table S2) is relatively higher in the case of the grapefruit IntegroPectin (Table S2). Although the C=O stretching band centered at ca. 1674–1690 cm^−1^ deriving from non-esterified hydrogenated acidic carbonyl and conjugated keto groups [10], as well as from carboxylic acid groups with strong H bonds [41], is a contributor to the spectral deconvolution for both IntegroPectins, this vibration was more represented in the lemon pectic sample (Tables S1 and S2), suggesting its more acidic nature compared to grapefruit IntegroPectin. On the other hand, the latter pectin features two distinctive IR contributions between 1640 and 1610 cm^−1^ (Table S2), corresponding to the presence of uracyl and phenyl moieties (1637 cm^−1^) deriving from the most abundant flavonoids adsorbed on the surface of the Citrus paradisi pectic polymer extracted via hydrodynamic cavitation [34], and to carboxylate groups (1613 cm^−1^), explaining the rapid solubilization of grapefruit IntegroPectin in water. Besides, given the strong IR absorbance characteristic of aromatic compounds in the 1595–1510 cm^−1^ range highlighted by the spectral deconvolution for both IntegroPectins (Figure 2B,C; Table S2), it is reasonable that these substances represent a large percentage of the total extracts obtained through the HC-based extraction process. 

Functional and technological properties of pectic polymers (i.e., gelling, stabilizing, and thickening behavior) are greatly affected by their degree of esterification (DE), namely the fraction of -COOH groups esterified with methanol [10,33,34]. We briefly remind that pectins with DE lower or higher than 50% are defined as low methoxy (LM) or high methoxy (HM) pectins, respectively [10]. Generally, HM-pectins form gels under acidic conditions [42,43], where an acid compound is required to suppress the dissociation of free -COOH groups and promote interaction between the polymeric chains [10], while these systems are easily produced by LM-pectins upon addition of divalent cations interacting with the free carboxylic groups within a broad range of pH values [10,43]. Thus, the Fidalgo–Ilharco equation (see Materials and Methods section) was used to assess the DE of commercial citrus pectin and the two IntegroPectins by calculating the integrated area values obtained either from (i) the IR bands for the former or (ii) after the spectral deconvolution by non-linear least-squares fitting of the 1800–1470 cm^−1^ region for the latter [10] (Table 1).

Low methoxy content (DE < 50%; Table 1) was detected for the three pectins studied, although the commercial citrus pectin showed a much larger DE value (i.e., 48%) compared to both lemon and grapefruit IntegroPectins. We ascribe this difference to the diverse extraction procedures. Indeed, the hydrolysis in hot acidic water typical of the conventional pectin extraction leads to the loss of hydrophilic RG-I chains in favor of HG “smooth” regions, which are better preserved by using microwave-assisted extraction [44] or, even more, acoustic cavitation [45]. In this regard, the low DE values observed for lemon (DE = 8%) and grapefruit (DE = 14%) IntegroPectin samples seem to indicate even greater preservation of RG-I chains through hydrodynamic cavitation with the respect to other extraction techniques, enhancing the applicative potential of these bioactive extracts. The diversity in DE values measured for lemon and grapefruit IntegroPectins (Table 1) further points to indicate the dependency of this parameter on the raw material itself (i.e., lemon or grapefruit peels), as also reported by Fidalgo et al. (2016) [10]. Indeed, likewise to the orange pectin analyzed by these authors, grapefruit IntegroPectin contains longer and more numerous hydrophilic RG-I regions in comparison to pectin from lemon, being comprised of randomly coiled molecular chains promoting a flexible conformation in solution and lower viscosity [46]. Additionally, proposing the first mechanism for pectin extraction from citrus peel driven by cavitation, Liu’s team showed that the molecular weight of cavitation-assisted extraction is lower than that of pectin obtained via the conventional hydrolytic route [45]. In other words, the molecular homogeneity of pectin obtained via cavitation is higher, when compared to conventional commercial pectin, but the molecular size is reduced. 

The group of intense and partially convoluted bands observed in the 1200−950 cm^−1^ region of the IR spectrum (Figure 2A; Table S1) is part of the so-called “fingerprint region” of polysaccharides comprising pectic polymers, whose IR spectral envelope depends on the crystallinity and conformation of polysaccharides themselves [47]. Overall, the deconvolution performed on the 1200–950 cm^−1^ region of the IR spectra (Figure 2D–F) showed high similarity, in terms of identified IR vibrational modes, between the commercial citrus pectin and the two analyzed IntegroPectins, with most of the absorption bands typical of the polygalacturonic acid [34] present within the pectins studied. Indeed, vibrational modes assigned to the skeletal and C−O−C stretching modes of both the pyranose ring and the glycosidic bond, as well as a combination of C−OH and C−C contributions deriving from the pyranose rings [10], were detected (Table S3). The spectral deconvolution in the 1200–950 cm^−1^ region (Figure 2D–F) revealed a higher integrated area attributable to IR vibrational modes typical of pectin ring and its glycosidic bonds (ca. 1140 cm^−1^) within the commercial citrus pectin with respect to the two IntegroPectins (Table S3), further indicating the presence of a larger amount of pectic polysaccharides in the former and a smaller yet comparable concentration in the case of both lemon and grapefruit IntegroPectins. Overall, the grapefruit IntegroPectin featured the highest signal attributable to uronic acid (1097–199 cm^−1^) and neutral sugars, such as arabinose and galactose (1067–970 cm^−1^; Table S3), in line with the results reported by La Cava et al. (2018) [34]. Indeed, galactose is highly represented in grapefruits as compared to other citrus species, resulting in the formation of short lateral chains [46]. Regardless of the similarities, grapefruit IntegroPectin showed an IR absorption band centered at ca. 1181 cm^−1^ attributable to both pectin ring vibration [48] and -HCC bending of flavonoids (i.e., narginin) [49], whose presence was suggested also by the detection of the symmetric -C=O stretching at ca. 1713 cm^−1^ (Tables S1 and S2), of the aromatic skeleton (1596–1510 cm^−1^; Tables S1 and S2) and the -CH_3_ rocking vibrations (ca. 1049 and 965 cm^−1^; Tables S1 and S3), as well as characteristic IR contributions in the 1400–1250 cm^−1^ range (Table S1), also detected for the lemon IntegroPectin. In this regard, the pink grapefruit features an elevated concentration of diverse flavonoids, including four glycosylated flavanones (isonaringin, naringin, hesperidin, and neohesperidin), two flavanone aglycones (hesperetin and naringenin), and four polymethoxylated flavones (isosinensetin, sinensetin, nobiletin, and tangeretin) [50]. Particularly, naringin (142 mg/g dry weight of peel) and isonaringin (11.85mg/g) are the predominant flavonoids in pink grapefruit peel, the concentration of the former being 2-to-4 times higher than in other citrus species [50]. With a concentration of 3.45 mg/g dry weight, finally, nobiletin (5,6,7,8,3′,4′-hexamethoxyflavone) is also particularly abundant in the peel of pink grapefruit [50]. 

The IR absorption bands detected at 829–833 cm^−1^ for the three pectin-based samples are attributable to α-glycosidic linkages [51], therefore suggesting the presence of these linkages between monomeric units of the pectic polymers [46]. On the other hand, the IR contributions centered in the 883–886 cm^−1^ range (Table S1) were ascribed to the characteristic C = CH_2_ out-of-plane bending mode of vinylidene groups deriving from terpenoids [10,52]. The vibrational modes of lemon and grapefruit IntegroPectins in the 860–650 cm^−1^ region were typical of phenol and polyphenol compounds [30,41,53], being only partially overlapped with IR contributions deriving from the pectic polymer identified in the commercial citrus pectin (Table S1).

**Figure 3:**
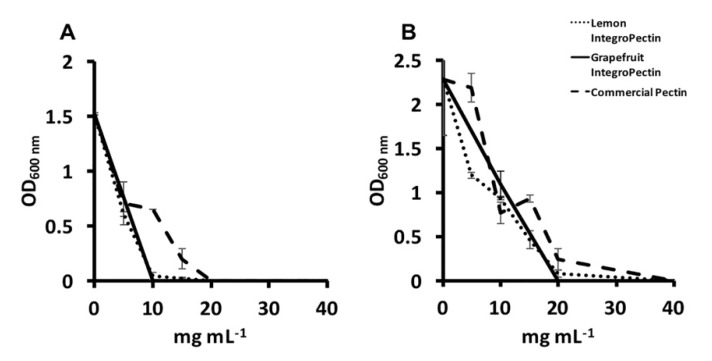
Minimal inhibitory concentration of either lemon or grapefruit IntegroPectin, as well as the commercial citrus pectin against (**A**) *Pseudomonas aeruginosa* and (**B**) *Staphylococcus aureus* strains.

The antimicrobial activity of either lemon or grapefruit IntegroPectin was tested against both *P. aeruginosa* ATCC 10145 and *S. aureus* ATCC 25923 indicator bacterial strains and compared to that achieved with the commercial citrus pectin. The former strain is commonly used as a standard laboratory testing control strain for drugs [54], while the latter is a clinical isolate resistant to antibiotics, including methicillin [55]. Grapefruit IntegroPectin could inhibit the growth of *P. aeruginosa* highlighting a minimal inhibitory concentration (MIC) (i.e., the lowest concentration of an antimicrobial that prevents the visible bacterial growth in a broth dilution susceptibility test) [56] value as low as 10 mg mL^−1^, while both the lemon IntegroPectin and commercial one exerted the same effect at MIC values of 20 mg mL^−1^ (Figure 3A). Compared to the Gram-negative bacterial strain, *S. aureus* displayed enhanced resistance towards the tested pectic materials, as highlighted by the doubled MIC value (20 mg mL^−1^) of grapefruit IntegroPectin, as well as from the need of 40 mg mL^−1^ of either lemon IntegroPectin or commercial citrus pectin to prevent the visible growth of *S. aureus* cells (Figure 3B).

**Figure 4:**
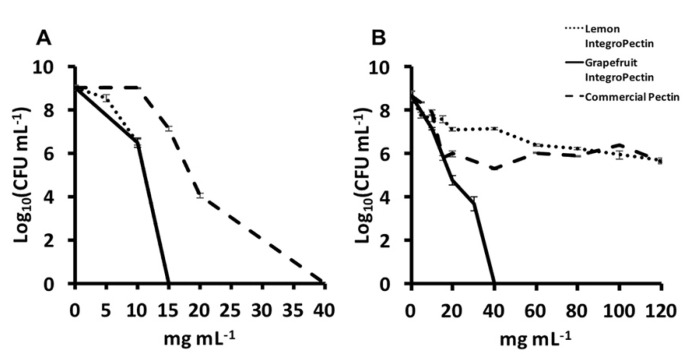
Minimal bactericidal concentration of either lemon or grapefruit IntegroPectin, as well as the commercial citrus pectin against (**A**) *Pseudomonas aeruginosa* and (**B**) *Staphylococcus aureus* strains.

A similar conclusion can be drawn for the evaluated amount of viable colony forming units (CFU) per mL of culture surviving the challenge exerted by the presence of each pectic material. Indeed, lemon and grapefruit IntegroPectins showed a powerful antimicrobial activity that completely killed the initial amount of inoculated *P. aeruginosa* cells at a concentration as low as 15 mg mL^−1^, likely due to flavonoids nobiletin and tangeretin capable of inhibiting the activities of key enzymes and impairing protein synthesis in *P. aeruginosa* [57]. The commercial citrus pectin exerted the same effect at a higher microbial bactericidal concentration (MBC) of 40 mg mL^−1^ (Figure 4A), as these flavonoids were present in far lower amounts. In the case of *S. aureus*, grapefruit IntegroPectin was the only pectic material that completely counteracted the thriving of the initial microbial load (Figure 4B), in agreement with the antibacterial action of grapefruit peel ethanol extract against Gram-positive bacteria [58]. 

In detail, the number of *S. aureus* in CFU mL^−1^ halved from 9 to 4.5, in terms of logarithmic units, in the presence of 20 mg mL^−1^ grapefruit IntegroPectin. It was enough to double to 40 mg mL^−1^ the concentration of grapefruit IntegroPectin to observe the bactericidal action against the Gram-positive bacterial strain (Figure 4B). Contrary to the great antimicrobial activity of the latter pectic material, the MBC value of both lemon IntegroPectin and commercial citrus pectin could not be determined. Even at 120 mg mL^−1^ (i.e., the highest concentration tested) these pectic materials did not show bactericidal action, even though, in agreement with what recently reported [12], they exerted a bacteriostatic effect as shown by the decrease of 3-log_10_ units in the number of CFUs mL^-1^ concerning unchallenged cultures (Figure 4B). The significance of these results, in light of forthcoming clinical trials, is even reinforced by the fact that the recommended inoculum size in the broth dilution method to determine the MIC of an antimicrobial substance is 5 × 10^5^ CFU mL^−1^ [59] while, in the present study, the culture broth was inoculated with a cellular load 1 order of magnitude higher (6 × 10^6^ CFU mL^−1^).

**Figure 5:**
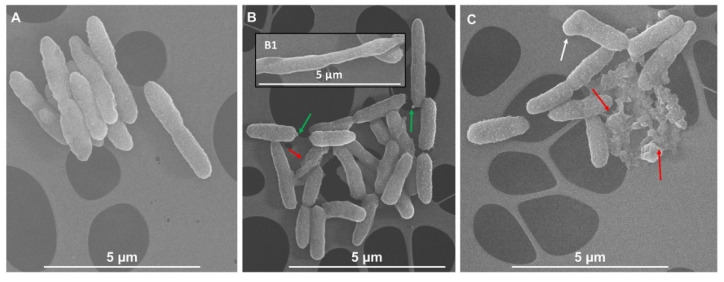
Scanning electron micrographs of unchallenged (**A**) or challenged *P. aeruginosa* cells with lemon (**B**) and/or grapefruit (**C**) IntegroPectins. The inlet **B1** shows an elongated and undivided cell. Nonorthodox cell morphology is indicated by white arrow; cell death events are underlined by red arrows, while green arrows highlight the presence of blebs of the cell envelope.

**Figure 6:**
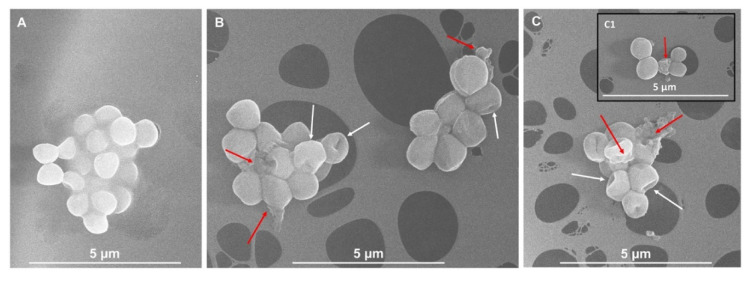
Scanning electron micrographs of unchallenged (**A**) or challenged *S. aureus* cells with lemon (**B**) and/or grapefruit (**C**) IntegroPectins. The inlet **C1** shows a collapsed coccus cell. Nonorthodox cell morphologies are indicated by white arrows; cell death events are underlined by red arrows.

Given the complex composition of IntegroPectins as compared to the commercial one highlighted by UV-vis and ATR-FTIR spectroscopic analyses, the superior antimicrobial activity against not only Gram-negative but also Gram-positive strains reasonably relies on a synergistic effect derived from the combined action of bioactive molecules (i.e., phenols, polyphenols, flavonoids, and terpenoids) and the pectic polysaccharides constituting this natural pectic extract, as opposed to commercial citrus pectins, which preferentially exert a bactericidal activity against Gram-negative bacteria only [60]. Among the numerous cellular targets of polyphenols, flavonoids, and terpenes [61], these compounds were shown to efficiently inhibit in vivo and in vitro the polymerization and the GTPase activity [62] of the filamenting temperature-sensitive mutant Z (FtsZ) protein (homolog to the eukaryotic tubulin), which is responsible for the bacterial cytokinesis [62,63], eventually leading to cell death. In this regard, the presence of either elongated bacterial cells or uncomplete division septa can be considered as a hallmark of an altered microbial morphology, underlining how the finely regulated cellular division process is perturbed. In the present case, upon exposure of *P. aeruginosa* and *S. aureus* cells for 2 h to either lemon (Figure 5C and Figure 6B) or grapefruit (Figure 5C and Figure 6C) IntegroPectin, nonorthodox cell morphologies (indicated by white arrows) were observed in comparison to unchallenged cells (Figure 5A and Figure 6A). Particularly, both IntegroPectins may affect the cell viability by hampering a proper cellular division, which is highlighted by the occurrence of either longer bacilli (Figure 5, inlet B1) or collapsed cocci (Figure 6B and inlet C1). Considering the presence of all the above-mentioned bioactive molecules in the IntegroPectin material tested, it is reasonable to suggest that one mode of action through which this new class of pectic materials could deploy antimicrobial action relies on the induction of defects during the cell division process, therefore determining cell death (indicated by red arrows). Moreover, we could speculate that the microbial oxidation of carbohydrates present in the pectic material itself under aerobic conditions can lead to the turning-off of the tricarboxylic acid (TCA) cycle with the consequent accumulation of pyruvate [64], which can be converted into acetic acid, the latter being secreted until the sugar catabolism is complete [65]. The moment bacterial cells start to uptake acetic acid, its high amount within the bacterial cytoplasm can tip the balance towards cell life versus death, as acetic acid can stimulate the murein hydrolase activity, compromising the cell viability [66,67]. Additionally, *P. aeruginosa* cells exposed to lemon IntegroPectin showed the occurrence of blebs at the cell envelope level (Figure 6B indicated by green arrows), which is the route most exploited by Gram-negative strains to secrete both insoluble and soluble molecules [68,69], as a stress response mechanism elicited by bacterial cells to the challenge represented by the IntegroPectin material. 

Indeed, this vesicular process might be even more pronounced by the partitioning of aromatic compounds present in the IntegroPectin samples in the nonpolar regions of the biological membrane, causing the alteration of its physiological fluidity analogously to the effect exerted by hydrophobic compounds [70,71,72]. It also relevant to this study, that recently scholars in Brazil reported the excellent bactericidal activity of terpineol, a terpene present in both lemon and grapefruit peel and seed, against *S. aureus* strains, with the hydroxyl-bearing terpineol interrupting cell division and altering the bacterial cell morphology [73].

## 3. Materials and Methods

### 3.1. UV-visible and Fourier Transform Infrared (FTIR) Spectroscopies of Commercial Citrus Pectin, Lemon, and Grapefruit IntegroPectins

UV-visible spectra were recorded in the 200–700 nm range for aliquots (1 mL) of commercial citrus pectin, lemon, and grapefruit IntegroPectins (2 mg mL^−1^) by using a Beckman DU 800 spectrophotometer (Beckman Coulter Life Sciences, Milan, Italy). 

FTIR spectra in attenuated total reflectance (ATR) mode of both lemon and grapefruit IntegroPectins were collected in the 40–4000 cm^−1^ range with a lateral resolution of 2 cm^−1^ and 200 scans by using an FTIR Bruker Vertex70 Advanced Research Fourier Transform Infrared Spectrometer (FTIR, Billerica, MA, USA) equipped with a Platinum ATR and a diamond crystal. 

The spectra were subsequently analyzed through the software OPUS (7.5), which was provided with the instrument, as well as OriginPro^®^ 2016 software. 

The degree of esterification (*DE*) of both lemon and grapefruit IntegroPectins was determined following the Fidalgo–Ilharco equation described elsewhere [10]:

(1) $$DE=\frac{\sum A_{v\left(C=O\right)ester}}{\sum A_{v\left(C=O\right)ester}+\sum A_{v\left(C=O\right)acid}+\sum A_{v_{as}\left(COO^{-}\right)}}$$

where *A* indicates the integrated area obtained after the spectra deconvolution by non-linear least-squares fitting (OriginPro^®^ 2016 software) of the 1800–1400 cm^−1^ region for both the analyzed samples. 

### 3.2. MBC and MIC Evaluation of Lemon and Grapefruit IntegroPectin

The antibacterial activity of both lemon and grapefruit IntegroPectins was assessed by determining their MBC and MIC values against both *P. aeruginosa* ATCC 10145 and *S. aureus* ATCC 25923 indicator strains. The MBC of IntegroPectins was established by inoculating stationary grown *P. aeruginosa* or *S. aureus* cells in Luria Bertani medium (hereafter named as LB and composed of 10 g sodium chloride, 10 g tryptone, and 5 g yeast extract per liter) amended with increasing concentrations (i.e., 5, 10, 15, 20, and 40 mg mL^−1^) of either lemon or grapefruit IntegroPectin; the initial concentration of the former was further increased to 60, 80, or 120 mg mL^−1^ only in the case of *S. aureus* cells. Bacterial cultures were then incubated for 24 h at 37 °C under mechanical shacking (180 rpm). The commercial citrus pectin (galacturonic acid ≥ 74.0%, dry basis, from Merck Life Science, Milan, Italy) was used as a comparison at concentrations corresponding to those of IntegroPectins, while non-challenged bacterial cells were incubated under the same conditions as a control. After 24 hours’ challenge, bacterial cultures were serially diluted and aliquots (20 µL) were spotted onto LB agar (15 g L^−1^) plates, which were recovered at 37 °C under static mode. The kill curves reporting the number of viable CFU mL^−1^ as a function of the challenges’ concentration are expressed in the logarithmic (Log_10_) scale with standard deviation (n = 3), as described elsewhere [74].

As for the MIC evaluation of IntegroPectins, at the end of the bacterial challenge (24 h), the optical density at 600 nm (OD_600_) of bacterial cultures was spectrophotometrically read by using a UV-visible spectrophotometer Jasco 7850 (Lecco, Italy, Jasco Europe). The experiment was performed in biological triplicate (n = 3) and the data are reported as means of the absorbance value with standard deviation.

All the reagents were purchased from Sigma-Aldrich (Milan, Italy).

### 3.3. Scanning Electron Microscopy (SEM) Imaging of Bacterial Cells Exposed to IntegroPectins

Stationary grown cells of either *P. aeruginosa* or *S. aureus* strains were independently inoculated 1% (*v*/*v*) on fresh LB medium and grew for 2 h prior to their exposure (for 2 additional hours) to 15 mg mL^−1^ of either lemon or grapefruit IntegroPectins. Then, bacterial cells were pelleted at 8000x *g* for 10 min, washed twice with sterile saline (0.9% *w*/*v*) solution, and resuspended in 2.5% (*v*/*v*) glutaraldehyde solution. The samples were stored overnight (ca. 18 h) at 4 °C to fix bacterial cells. The day after, bacterial cells were pelleted as described above, being then dehydrated through three washing steps (10 min each) with increasing concentration (30, 40, 50, 60, 70, 80, 90% *v*/*v*, and absolute) of ice-cold ethanol. Right after, the cells were opportunely diluted and deposited onto carbon-coated copper grids (300 mesh) and observed through an FEG-SEM FEI versa 3D microscope, using an accelerating voltage of 10kV, as previously described [75].

## 4. Conclusions

We have discovered a new water-soluble bactericidal agent suitable for the treatment of polymicrobial infections, consisting of lemon and grapefruit pectins extracted, respectively, from waste lemon and waste grapefruit peels via hydrodynamic cavitation in water. Called IntegroPectin, this bioactive material comprises pectic polysaccharides with a low degree of esterification and abundant “hairy” regions (preserved during the cavitation-based extraction) with plentiful citrus terpenes and flavonoids adsorbed on the polysaccharide. Although pectic materials preferentially exert a bactericidal activity against Gram-negative strains, the IntegroPectins could inhibit the thriving of both *P. aeruginosa* and *S. aureus* cells. Morphological changes of bacterial cells exposed to IntegroPectins were detected, suggesting a mode of action based on the induction of defects during the cell division process. Accordingly, the mechanism of action may rely on a synergistic effect elicited by bioactive molecules present in the IntegroPectins, opposed to commercial citrus pectins, in combination with the cavitation-derived pectin rich in RG-I regions, particularly in the case of grapefruit IntegroPectin containing longer and more numerous hydrophilic RG-I regions in comparison to pectin from lemon [45]. Reporting the outcomes of the SEM investigation of the interaction of IntegroPectin in solution and the strains of ubiquitous *S. aureus* and *P. aeruginosa*, this study establishes the proof-of-concept of the superior antibacterial activity of crude citrus IntegroPectin compared to conventional citrus pectin. Specifically, a qualitative and quantitative analysis of the flavonoids and terpenes present in the lemon and in the grapefruit IntegroPectin will allow for the nature of those biomolecules and other possible polyphenols to be established. In the era of antimicrobial resistance, the natural IntegroPectin extracts can constitute valid alternatives to antibiotics, due to their non-cytotoxicity and multiple modes of action against bacteria, making the development of drug resistance unlikely. Further studies aimed at expanding the scope and application of this new biomaterial are underway in our Laboratories.

## Figures and Tables

**Table 1:** Degree of esterification (DE) for both lemon and grapefruit pectin.

Equation Member	Commercial Citrus Pectin	Lemon IntegroPectin	Grapefruit IntegroPectin
∑A_ν(C = O)ester_	7.80	1.29	1.35
A_ν(C = O)acid_ + A_νas(COO-)_	0 + 8.38	14.53 + 0	8.01 + 1.55
DE (%)	48	8	14

## Supplementary Materials

The following are available online at https://www.mdpi.com/2079-6382/9/9/586/s1. Table S1: ATR-FTIR absorption bands of grapefruit and lemon pectin and their attribution (observed maxima). Table S2: Results of the spectral deconvolution by non-linear least-squares fitting of the 1800–1470 cm^−1^ region. Table S3: Results of the spectral deconvolution by non-linear least-squares fitting of the 1200–950 cm^−1^ region.
