# Supplementary material for: A New Water-Soluble Bactericidal Agent for the Treatment of Infections Caused by Gram-Positive and Gram-Negative Bacterial Strains
Source: Antibiotics (Basel). 2020 Sep 8;9(9):586. doi: 10.3390/antibiotics9090586 (PMC7558503; doi:10.3390/antibiotics9090586)
Supplement: Supplementary file 1 [file antibiotics-09-00586-s001.pdf]

Supplementary information for

A New Water-Soluble Bactericidal Agent for the Treatment of Infections Caused by Gram-Positive and Gram-Negative Bacterial Strains

Alessandro Presentato [1], Elena Piacenza [1,2], Antonino Scurria [3], Lorenzo Albanese [4], Federica Zabini [4], Francesco Meneguzzo [4], Domenico Nuzzo [5], Mario Pagliaro [3,*], Delia Chillura Martino [1,2], Rosa Alduina [1,*] and Rosaria Ciriminna [3]

[1] Department of Biological, Chemical, and Pharmaceutical Science and Technology (STEBICEF), University of Palermo, Viale delle Scienze, 90128 Palermo, Italy; alessandro.presentato@unipa.it (A.P.); elena.piacenza91@gmail.com (E.P.); delia.chilluramartino@unipa.it (D.C.M.)

[2] National Interuniversity Consortium of Materials Science and Technology (INSTM), Via G. Giusti 9, 50121 Firenze, Italy; elena.piacenza91@gmail.com (E.P.); delia.chilluramartino@unipa.it (D.C.M.)

[3] Istituto per lo Studio dei Materiali Nanostrutturati, CNR, via U. La Malfa 153, 90146 Palermo, Italy; antonino.scurria@ismn.cnr.it (A.S.); rosaria.ciriminna@cnr.it (R.C.)

[4] Istituto per la Bioeconomia, CNR, via Madonna del Piano 10, 50019 Sesto Fiorentino, FI, Italy; lorenzo.albanese@cnr.it (L.A.); federica.zabini@cnr.it (F.Z.); francesco.meneguzzo@cnr.it (F.M.)

[5] Istituto per la Ricerca e l'Innovazione Biomedica, CNR, via U. La Malfa 153, 90146 Palermo, Italy; domenico.nuzzo@cnr.it

* Correspondence: mario.pagliaro@cnr.it (M.P.); valeria.alduina@unipa.it (R.A.)

Received: 4 August 2020; Accepted: 7 September 2020; Published: date

Table S1. ATR-FTIR absorption bands of grapefruit and lemon pectin and their attribution (observed maxima).

**$\tilde{\nu}$ (cm^{-1})**			**Vibrational mode**	**Identification**
**Commercial citrus pectin**	**Lemon IntegroPectin**	**Grapefruit IntegroPectin**		
3347			ν (OH)	Polysaccharides [1,2]; water [1,2]
	3295	3293	ν (OH)	Polysaccharides [1,2]; water [1,2]; polyphenols [3]
2924	2925	2931	ν_{as} (CH_2); ν_{as} (CH_3); ν (CH)	Pectin backbone [1]; arabinose and galactose [1]
	2865	2873	ν_s (CH_3); ν (CH)	Pectin backbone [1]; pyranose rings [1]
2675(s)	2671(s)		ν (OH)	Free carboxylic acids [1,2]
		2658(s)	ν (OH)	Free carboxylic acids [1,2]
	2502(s)	2499(s)	ν (CO)O-H, satellite	Carboxylic acid dimers [1,2]
1737			ν $(C{=}O)_{ester}$	Methyl esterified carboxylic groups of galacturonic acid [1,2,4–7]
	1713	1715	ν_s (C=O)	Carboxylate and nonconjugated keto groups of carotenoids [1,2,11,12], phenols and flavonoids [3,8-9]
	1674(s)		ν $(C{=}O)_{acid}$	Nonesterified hydrogenated acidic carbonyl and conjugated keto groups [1]; formyl groups of carotenoids [8]; carboxylic acid groups with strong H bonds [10]
		1631(s)	δ (H_2O); ν (C=C); ν (C-C); ν (C=C)	Phenyl and uracyl groups [7]; water [1]; phenolic acids [3–9]
1607			ν_{as} (COO^-); ν (C-C)	Carboxylate groups of polygalacturonic acid [1,7,11,12]
	1594	1596	ν (C=O)	Aromatic skeleton and keto groups [3,9,13–15]
		1578(s)	ν (C=C); ν (C=O)	Aromatic skeleton and keto groups [3,9,13–15]
	1512	1518	δ_{ip} (CH); ν (C=C)	Phenyl rings [3,9,15]; carotenoid compounds [8]
1459(s)			δ_{as} (CH_3); δ (CH2); ν (C=C); ν_{as} (COO^-)	Pectin backbone [12–16]; aromatic compounds [9,17–19]
1441	1436(s)	1438(s)	δ_{as} (CH_3); ρ (CH_2) 1st overtone; ν (C-C)	Aromatic compounds [3,9,16-18]; ester methyl groups in galacturonic and rhamnose rings of pectin [1,2,11,12,19]
1402	1397	1399	δ(COH)COOH; ν_s (COO^-); δ_{ip} (CH); δ_s (CH_3)	Methyl groups [3,4]; ring vibration [3,9,12]; carboxylate pectin ester groups [2–4]
1361	1367	1367	δ_s (CH_2); δ_s (CH_3); β_s (CH_3); δ (OH); δ_{ip} (COH); δ_{op} (CH_3); δ_s (CH_3)	Ester methyl groups in galacturonic and rhamnose rings of pectin [1,2,12,16]; flavonoids [9,17–19]

$\tilde{\nu}$ (cm^{-1})			Vibrational mode	Identification
1328	1327	1331	δ (CH); ν_s (COO$^-$); ω (CH$_2$); δ_{ip} (C-O-H)	Pyranose in pectic ring [2,9,20]; metoxyphenolic substitutions [12]; alcohol hydroxyl groups in pyranose ring [1]
		1296	ρ_s (CH); ν (CO-O); ν_{ip} (OH)	Aromatic ethers [9,12,17–19]
1264	1261(s)		β (OH); ν (C-O-C)	Esters; hydroxyl groups of polysaccharides [1,3,12,16,19,20]
		1250(s)	ν (C-O)	Polyols (hydroxyflavonoids) [16–19]
1222	1222	1225	ν (C(CH$_3$)$_2$); ν (CO-O); ν_{ip} (OH); δ_{ip} (C-O-H)	Aromatic ethers [3,8,9,17–20]; metoxyphenolic substitutions [8]; alcohol hydroxyl groups in pyranose ring [1,2]
		1200(s)	ν (C(CH$_3$)$_2$); ν (C-C)	cyclic C-C bonds in the pectin ring [1,2,4,5,11,12,16]; flavonoids [3,16–19]
		1176	ρ_{as} (CH); ν (C-O); ν (C-C); δ (HCC)	cyclic C-C bonds in the pectin ring [1,2,4,5,11,12,16]; flavonoids [3,16-19]
1142	1142	1140	ν (C-O-C); ν (C-C); ν_{as} (O-C-O)	Glycosidic bond in polysaccharide ring [1,2,11,12,20]; cyclic C-C bonds in the pectin ring [7,12]
1094	1096	1096	ν (C-O); ν (C-OH); ν (C-O-C); ν (C-C)	Pyranose and glycoside [1,2,15,20]; pectin ring [2,11,12,19]; uronic acid [3,7]
1072	1070	1067(s)	ν (C-O); ϱ (CO); ν (C-O-C); ν (C-C) ν (C-OH)	Pyranose and glycoside [1,2,12,19]; arabinose and galactose [7]
1047	1046	1045	ν (C-O); ρ (CO); ν (C-O-C); ν (C-OH); ν (C-C); ρ (CH$_3$)	Pyranose and glycoside [1,2,11,12,19]; arabinose and galactose [7]; flavonoids [3,9,15,19]
1013	1010	1012	ν (C-C); ν (C-O)	Polysaccharides [2,3,7,20]; pectin (C2-C3, C2-O2, C1-O1) [12,19]; uronic acid [7]
	966	970	γ (=CH); ρ (CH$_3$); ν(C=C)$_{trans}$;	Polysaccharides [1-3,7,20]; arabinose and galactose [7]; flavonoids [3,9,15–19]
954(s)			δ (C=O); δ (CCH); δ (COH)	Polysaccharides in pectin [1–3,7,20]; carotenoids [8]
914	924	919	ρ (CH$_3$); α-anomeric linkage; δ_{op} (=CH)$_{trans}$; β (Ph); τ (HCC)	Ester methyl groups [1,12,20]; glucose and fructose [1,2,12]; phenyl moieties [3,9,15–18]; pectin [2,6,19]; flavonoids [3,9,15–19]
	909		ν (C-C); δ_{op} (CH); δ (CCH); δ (COH)	Aromatic compounds [3,9,15–19]; pectins [1,2,3,6,19]

$\tilde{\nu}$ (cm^{-1})			Vibrational mode	Identification
883	883	886	β (CH); δ (CCH); δ (COH); γ (=CH); δ_{op} (C=CH$_2$)	Methylene groups[1,12,20]; vinilydiene groups of terpenoids [21]
		864(s)	ρ (CH$_2$); δ_{ip} (CH); ρ (CH$_2$); β (C-C$_{ring}$)	Pyranose [1,2,11,12,19]; phenols [3,9,15–19]
845			ρ (CH$_2$)	Pectin
829	832	831	γ (OH); δ_{op} (CH)	Six-membered ring of polyphenols [3,9,15,19]; *α*-glycosidic linkages [7]; phenolic compounds [3,9,15,19]
805	805	812	ρ (CH$_2$); δ_{ip} (C-H); β_{op} (CH)	Pyranose [1,2,11,12,19]
780(s)	783	779(s)	ω (CH); ρ (CH$_2$); δ_{ip} (C-H); γ (COH); δ_{op} (=CH) *cis*	Pyranose [1,2,11,12,19]; six-membered ring of polyphenols [3,9,15,19]
758	761	759	δ_{op} (=CH)$_{cis}$; breath	Breathing ring [1,12]
743			β_{op} CH$_{cis}$; ϱ_{ip} CH$_2$	
	709	712	ρ (CH$_2$); γ (COH); δ_{op} (=CH); δ_{op} (=CH)cis	six-membered ring of polyphenols [3,9,15,19]; vibrations of pyranoid ring [1,2,11,12,19]
		703	δ_{op} (=CH)	Pectin [1,2,3,6,19]
	684	686(s)	ω (C=O); δ_{op} (=CH); ν_s (C-O-C)	Glycoside linkage [1,2]; acidic pectins [1,2,3,6,19]
	659	660	β (C-C-O); γ (C-O)	Phenols [3,9,15,19]

$\tilde{\nu}$ (cm^{-1}) = wavenumber; ν = stretching, δ = bending/scissoring, ϱ = rocking, β = deformation modes, ω = wagging, breath = breathing, γ = out of plane ring vibrations, τ = twisting; as and s = asymmetric and symmetric; ip and op = in plane and out of plane, respectively.

Table S2. Results of the spectral deconvolution by non-linear least-squares fitting of the 1800-1470 cm^{-1} region.

Lemon IntegroPectin			Grapefruit IntegroPectin			Vibrational mode	Reference
**$\tilde{\nu}$ (cm^{-1})**	**w**	**A**	**$\tilde{\nu}$ (cm^{-1})**	**w**	**A**		
1750	20.6	1.29	1750	21.2	1.35	ν (C=O)$_{ester}$	[1,2,4–7]
1723	39.5	7.65	1722	40.3	8.87	ν_s (C=O)$_{carboxylate\ and\ nonconj.\ keto\ groups}$	[1–3,8–9,11,12]
1689	71.5	14.53	1678	58.3	8.01	ν (C=O)$_{acid}$	[1,8,10]
			1637	23.5	2.69	δ (H$_2$O); ν (C=C)$_{uracyl}$; ν (C-C)$_{phenyl}$	[1,3–9]
			1613	22.7	1.55	ν_{as} (COO$^-$)$_{carboxylate}$	[1,7,11,12]
1594	67.5	22.82	1591	55.9	17.90	ν (C=O)$_{aromatic\ skeleton\ and\ keto\ groups}$	[3,9,13–15]
1511	19.5	0.666	1516	15.7	0.77	δ_{ip} (CH); ν (C=C)	[3,8,9,15]

$\tilde{\nu}$ (cm^{-1}) = wavenumber; w = width; A = integrated area.

Table S3. Results of the spectral deconvolution by non-linear least-squares fitting of the 1200-950 cm^{-1} region.

**Commercial citrus pectin**			**Lemon IntegroPectin**			**Grapefruit IntegroPectin**			**Vibrational mode**	**Reference**
**$\tilde{\nu}$ (cm^{-1})**	**w**	**A**	**$\tilde{\nu}$ (cm^{-1})**	**w**	**A**	**$\tilde{\nu}$ (cm^{-1})**	**w**	**A**		
						1181	18.17	0.78	ρ_{as} (CH); ν (C-O); ν (C-C)$_{pectin\ ring}$; δ(HCC)$_{flavon.}$	[1–5,11,12,16–19]
1145	30.85	4.30	1141	25.90	2.58	1139	23.81	2.68	ν (C-O-C)$_{glycosidic\ bond}$; ν (C-C)$_{pectin\ ring}$	[1,2,7,11,12,20]
1097	30.75	8.75	1099	27.32	8.22	1098	30.93	9.46	ν (C-O); ν(C-O-C); ν (C-OH); ν (C-C)	[1–3,7,11,12,15,19,20]
1069	20.00	4.90	1070	21.30	6.42	1067	26.12	9.64	ν (C-O); ρ (CO); ν (C-O-C); ν (C-OH); ν (C-C)	[1,2, 7,12,19];
1049	17.11	3.20	1047	18.47	5.20	1045	20.37	6.53	ν (C-O); ρ (CO); ν (C-O-C); ν (C-OH); ν (C-C); ρ (CH_3)$_{flavon.}$	[1–3, 7,9,11,12,19]
1016	34.70	14.10	1012	36.87	17.60	1015	36.18	20.90	ν (C-C); ν (C-O)	[2,3,7,12, 19,20]
966	45.52	8.50	967	28.70	5.30	970	34.61	8.92	γ (=CH); C=C *trans*; ρ (CH_3)$_{flavon.}$	[1–3,7,9, 15–20]

$\tilde{\nu}$ (cm^{-1}) = wavenumber; w = width; A = integrated area.

References

1. Fidalgo, A.; Ciriminna, R.; Carnaroglio, D.; Tamburino, A.; Cravotto, G.; Grillo, G.; Ilharco, L.M.; Pagliaro, M. Eco-friendly extraction of pectin and essential oils from orange and lemon peels. *ACS Sustainable Chem. Eng.* **2016**, *4*, 2243–2251.
2. Synytsya, A.; Copikova, J.; Matejka, P.; Machovic, V. Fourier transform Raman and infrared spectroscopy of pectins. *Carbohydr. Polym.* **2003**, *54*, 97–106.
3. Heredia-Guerrero, J.A.; Benitez, J.J.; Dominguez, E.; Baye, I.S.; Cingolani, R.; Athanassiou, A.; Heredia, A. Infrared and Raman spectroscopic features of plant cuticles: a review. *Front. Plant. Sci.* **2014**, *5*, 305.
4. Manrique, G.D.; Lajolo, F.M. FT-IR spectroscopy as a tool for measuring degree of esterification in pectins isolated from ripening papaya fruit. *Postharvest Biol. Tec.* **2002**, *25*, 99–107.
5. Kyomugasho, C.; Christiaens, S.; Shpigelman, A.; Van Loey, A.M.; Hendrickx, M.E. FT-IR spectroscopy, a reliable method for routine analyses of the degree of methyl esterification of pectin in different fruit- and vegetable-based matrices. *Food Chem.* **2015**, *176*, 82–90.
6. Wang, W.; Ma, X.; Jiang, P.; Hu, L.; Zhi, Z.; Chen, J.; Ding, T.; Ye, X.; Liu, D. Characterization of pectin from grapefruit peel: a comparison of ultrasound-assisted and conventional heating extractions. *Food Hydrocoll.* **2016**, *61*, 730–739.

7. La Cava, E.L.; Gerbino, E.; Sgroppo, S.C.; Gomez-Zavaglia, A. Characterization of pectins extracted from different varieties of pink/red and white grapefruits [*Citrus paradisi* (Macf.)] by thermal treatment and thermosonication. *Food Chem.* **2018**, *83*, 1613–1621.
8. Lorand, T.; Deli, J.; Molnar, P.; Toth, G. FT-IR study of some carotenoids. *Helv. Chim. Acta*, **2002**, *85*, 1691–1697.
9. Ricci, A.; Olejar, K.J.; Parpinello, G.P.; Kilmartin, P.A.; Versari, A. Application of Fourier transform infrared (FT-IR) spectroscopy in the characterization of tannins. *Appl. Spectrosc. Rev.* **2015**, *50*, 407–442.
10. Espana, L.; Heredia-Guerrero, J.A.; Segado, P.; Benitez, J.J.; Heredia, A.; Dominguez, E. Biochemical properties of the tomato (*Solanum lycopersicum*) fruit cuticle during development are modulated by changes in the relative amounts of its components. *New Phytol.* **2014**, *202*, 790–802.
11. Aburto, J.; Moran, M.; Galano, A.; Torres-Garcia, E. Non-isothermal pyrolysis of pectin: a thermochemical and kinetic approach. *J. Anal. Appl. Pyrol.* **2015**, *112*, 94–104.
12. Bichara, L.C.; Alvarez, P.E.; Fiori Bimbi, M.V.; Vaca, H.; Gervasi, C.; Brandan, S.A. Structural and spectroscopic study of a pectin isolated from citrus peel by using FTIR and FT-Raman spectra and DFT calculations. *Infrared Phys. Techn.* **2016**, *76*, 315–327.
13. Zeier, J.; Schreiber, L. Fourier transform infrared-spectroscopic characterization of isolated endodermal cell walls from plant roots: chemical nature in relation to anatomical development. *Planta.* **1999**, *209*, 537–542.
14. Rashid, T.; Kait, C.F.; Murugesan, T. A "Fourier transformed infrared" compound study of lignin recovered from a formic acid process. *Procedia Eng.* **2016**, *148*, 1312–1319.
15. Zeier, J.; Schreiber, L. Fourier transform infrared-spectroscopic characterization of isolated endodermal cell walls from plant roots: chemical nature in relation to anatomical development. *Planta.* **1999**, *209*, 537–542.
16. Sene, C.F.; McCann, M.C.; Wilson, R.H.; Grinter, R. Fourier-transform Raman and Fourier-transform infrared spectroscopy: an investigation of five higher plant cell walls and their components. *Plant Phys.* **1994**, *106*, 1623–1631.
17. Suresh, R.; Balakumar, R.; Krishnakumar, N.; Saleem, H.; Subashchandrabose, S. Structural geometry, vibrational and electronic spectra investigation on narginin molecules using experimental and density functional theory calculation. *Int. J. Mol. Theor. Phy.* **2018**, *2*, 1–22.
18. Tracanna, M.I.; Romano, E.; Raschi, A.B.; Fortuna, A.M.; Brandan, S.A. Structural and FT-IR studies of phenolic 2,8-dihydroxy-7H-furo [2,3-f]chromen-7-one derivative isolated from *Tibouchina paratropica*. *J. Mater. Environ. Sci.* **2019**, *10*, 818–831.
19. Schulz, H.; Baranska, M. Fruits and vegetables. In *Infrared Spectroscopy for Food Quality Analysis and Control*, Sun, D.W., Ed. Academic Press, London, UK, 2009; pp. 321-353.
20. Copikova, J.; Synytsya, A.; Cerna, M.; Kaasova, J.; Novotna, M. Application of FT-IR spectroscopy in detection of food hydrocolloids in confectionery jellies and food supplements. *Czech. J. Food Sci.* **2001**, *19*, 51-56.
21. Mitzner, B.M.; Theimer, E.T. β-Terpinene and β-Pellandrene from the pyrolysis of Sabinene. *J. Org. Chem.* **1962**, *27*, 3359.
